# Plant peroxisomes are degraded by starvation-induced and constitutive autophagy in tobacco BY-2 suspension-cultured cells

**DOI:** 10.3389/fpls.2014.00629

**Published:** 2014-11-18

**Authors:** Olga V. Voitsekhovskaja, Andreas Schiermeyer, Sigrun Reumann

**Affiliations:** ^1^Department of Plant Biochemistry, Albrecht-von-Haller-Institute for Plant Sciences, Georg-August-Universität GöttingenGöttingen, Germany; ^2^Komarov Botanical Institute, Russian Academy of Sciences, Laboratory of Plant Ecological PhysiologySaint Petersburg, Russia; ^3^Abteilung Pflanzenbiotechnologie, Fraunhofer-Institut für Molekularbiologie und Angewandte OekologieAachen, Germany; ^4^Institute for Mathematics and Natural Sciences, Faculty of Science and Technology, Centre for Organelle Research, University of StavangerStavanger, Norway; ^5^Faculty of Mathematics, Informatics and Natural Sciences, Biocentre Klein Flottbek, University of HamburgHamburg, Germany

**Keywords:** peroxisome, autophagy, pexophagy, tobacco BY-2 cells, organelle degradation, cellular peroxisome pool

## Abstract

Very recently, autophagy has been recognized as an important degradation pathway for quality control of peroxisomes in *Arabidopsis* plants. To further characterize the role of autophagy in plant peroxisome degradation, we generated stable transgenic suspension-cultured cell lines of heterotrophic *Nicotiana tabacum* L. cv. Bright Yellow 2 expressing a peroxisome-targeted version of enhanced yellow fluorescent protein. Indeed, this cell line model system proved advantageous for detailed cytological analyses of autophagy stages and for quantification of cellular peroxisome pools under different culturing conditions and upon inhibitor applications. Complementary biochemical, cytological, and pharmacological analyses provided convincing evidence for peroxisome degradation by bulk autophagy during carbohydrate starvation. This degradation was slowed down by the inhibitor of autophagy, 3-methyladenine (3-MA), but the 3-MA effect ceased at advanced stages of starvation, indicating that another degradation mechanism for peroxisomes might have taken over. 3-MA also caused an increase particularly in peroxisomal proteins and cellular peroxisome numbers when applied under nutrient-rich conditions in the logarithmic growth phase, suggesting a high turnover rate for peroxisomes by basal autophagy under non-stress conditions. Together, our data demonstrate that a great fraction of the peroxisome pool is subject to extensive autophagy-mediated turnover under both nutrient starvation and optimal growth conditions. Our analyses of the cellular pool size of peroxisomes provide a new tool for quantitative investigations of the role of plant peroxisomes in reactive oxygen species metabolism.

## INTRODUCTION

Plant peroxisomes perform many important functions including metabolism of reactive oxygen species (ROS), photorespiration, lipid metabolism, synthesis of plant hormones, polyamine metabolism, and ureate catabolism (Hayashi and Nishimura, 2003; del Rio et al., 2006; Reumann and Weber, 2006; Graham, 2008; Moschou et al., 2008; Kaur et al., 2009; Palma et al., 2009). Proteomic methodology recently allowed the recognition of additional metabolic functions, including, for instance, in the biosynthesis of phylloquinone (Babujee et al., 2010). Plant peroxisomes also play essential roles in photomorphogenesis, embryogenesis, and seed germination and in plant pathogen defense mechanisms (Kaur et al., 2009; Hu et al., 2012). Peroxisome biogenesis has been studied in considerable detail both mechanistically and at the molecular level. More than 30 PEXs have been cloned and functionally been characterized in fungi, mammals, and plants (for review, see Kaur et al., 2009; Ma and Subramani, 2009; Ma et al., 2011; Hu et al., 2012). Contrary to plant peroxisome biogenesis, our knowledge about mechanisms of peroxisome degradation in plant cells has remained scarce.

Studies in fungi and mammals demonstrated that peroxisomes are degraded by autophagy (Farre and Subramani, 2004; Dunn et al., 2005). Autophagy is a catabolic process for cellular remodeling and macromolecule recycling to tolerate extensive phases of nutrient starvation and to eliminate superfluous and dysfunctional cell organelles. Autophagy ultimately leads to degradation of cytoplasmic structures such as single proteins, protein complexes, and entire organelles in the acidic central vacuole (yeast, plants) or lysosome (mammals). Two major autophagic pathways have been described, macro- and microautophagy (Bassham, 2007, 2009). Macroautophagy, which is the major and best characterized mechanism, is initiated in the cytoplasm with the formation of cup-shaped membranes termed the preautophagosomal structure. The elongating membranes enclose the material to be degraded in double-membrane autophagosomes that subsequently fuse with the vacuole. The outer membrane of the autophagosome fuses with the tonoplast and releases its content, referred to as the autophagic body, into the vacuolar lumen (Li and Vierstra, 2012; Liu and Bassham, 2012). By contrast, in microautophagy, cytosolic proteins or entire cell organelles are directly engulfed by the vacuole by invagination of the tonoplast.

Macroautophagy, hereafter referred to as autophagy, has been also reported to exist in plants, where the mechanism is involved in various developmental processes, including the formation of the vegetative and protein storage vacuoles and senescence (Moriyasu and Hillmer, 2000). Plant autophagy is induced by various types of nutrient starvation. Degradation of non-essential cellular components liberates energy and catabolic intermediates that can be recycled to maintain basal levels of cellular metabolism. Plant autophagy is also important to cope with adverse environmental stress conditions by removal of damaged dysfunctional structures. During senescence in individually darkened leaves, chloroplasts were shown to be degraded by autophagy (Wada et al., 2009). *Arabidopsis* loss-of-function mutants of genes homologous to yeast Autophagy-related (Atg) genes are generally viable but exhibit early senescence (for review, see Thompson and Vierstra, 2005; Bassham, 2007, 2009; Reumann et al., 2010; Li and Vierstra, 2012).

Very recently, the first reports on plant pexophagy, or selective degradation of peroxisomes by autophagy have been published. As deduced from pharmacological studies inhibiting autophagy and genetic experiments using *atg7* mutants, peroxisomes in *Arabidopsis thaliana* hypocotyls are turned over by autophagy during seedling growth (Kim et al., 2013, 2014). In leaves of *atg5* gene knockout *Arabidopsis thaliana* mutants, peroxisomes accumulated and contained elevated levels of insoluble inactive catalase (CAT) (Yoshimoto et al., 2014). In a forward genetic screen for *Arabidopsis* mutants altered in peroxisomal positioning, Shibata et al. (2013) identified three *Arabidopsis thaliana* mutants that contained aggregated peroxisomes and whose gene defects were identical to autophagy mutants (*atg2*, *atg18a,* and *atg7*). The number of peroxisomes was increased, and the aggregated peroxisomes, which co-localized with the autophagosome marker, ATG8, contained high levels of inactive CAT indicative of damaged peroxisomes. Hence, the most recent data provided evidence that autophagy is crucial for quality control mechanisms for peroxisomes in *Arabidopsis* (Kim et al., 2013, 2014; Shibata et al., 2013; Yoshimoto et al., 2014).

Contrary to whole plants, suspension-cultured tobacco (*Nicotiana tabacum*) BY-2 cells offer several advantages for autophagic studies, including their accessibility to inhibitors and small fluorescent molecules and the ability to induce autophagy by sucrose starvation. For instance, autophagy can be blocked by 3-MA in BY-2 cells (Takatsuka et al., 2004). To establish suspension-cultured BY-2 cells as a model system for studies of plant pexophagy and characterize the degradation mechanism in greater detail, we labeled peroxisomes in tobacco BY-2 cells with EYFP-SKL. By comprehensive biochemical and cytological analyses we demonstrate in this study that peroxisomes in BY-2 cells are degraded by autophagy under nutrient starvation. We furthermore show that even under optimal, nutrient-sufficient growth conditions and in exponential growth phase, peroxisomes are subjected to autophagy-mediated turnover, indicating a major physiological need for replacement of most likely dysfunctional peroxisomes in plants.

## MATERIALS AND METHODS

### PLANT MATERIAL AND GENERATION OF STABLE TRANSGENIC BY-2 CELL LINES

**FIGURE 1:**
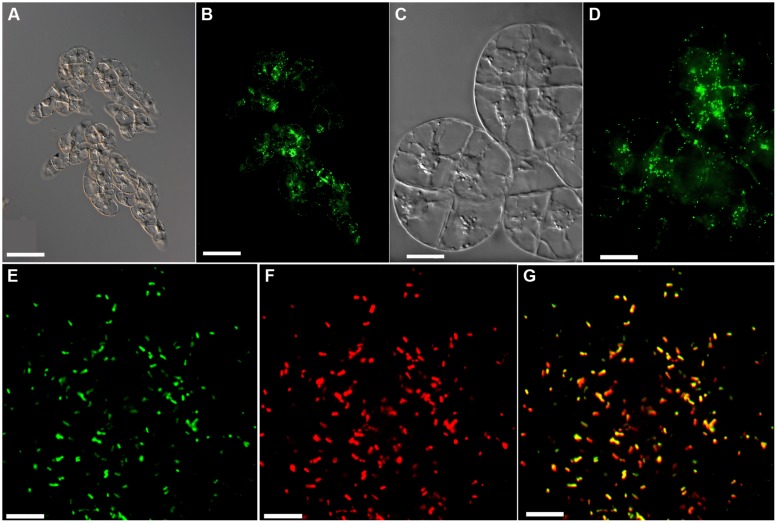
**Fluorescent labeling of peroxisomes by EYFP-SKL. (A–D)** Live-cell imaging of BY-2 transformants expressing *CaMV35S::*EYFP-SKL with Nomarski optics **(A,C)** or epifluorescence showing peroxisomes labeled by EYFP-SKL **(B,D)**. CLSM imaging of EYFP **(E),** CAT immunofluorescence **(F),** and the merge of both **(G)**. Scale bar 100 μm **(A,B)**, 20 μm **(C,D)**, and 4 μm **(E–G)**. CLSM confirmed that the strong targeting signal of the *Arabidopsis* photorespiratory enzyme, hydroxypyruvate reductase (SKL), directed the fluorophore to peroxisomes with high efficiency, without any cytosolic background fluorescence when grown under nutrient-sufficient culturing conditions (**E,F** compare with images on **B,D** obtained by epifluorescence microscopy where some “cytosolic” background represents the EYFP fluorescence emitted by out-of-focus peroxisomes).

Tobacco (*N. tabacum*) BY-2 suspension cells were stably transformed by *Agrobacterium*-mediated transformation (An, 1985) to express a peroxisome-targeted version of EYFP from the CaMV 35S promoter. For peroxisome targeting the reporter protein was extended C-terminally by the last ten amino acid residues (KALGLPVSKL) of *Arabidopsis* hydroxypyruvate reductase (Genbank reference sequence NP_176968, At1g68010) using the primers EYFP-for (*Nco*I, 5′-AAGTCCATGGTGAGCAAGGGCGAGGA-3′) and EYFP-PTD_HPR_-rev (*Xba*I, 5′-TATATCTAGATCATAGCTTCGAAACAGGCAATCCTAAGGCCTTCTTGTACAGCTCGTCCATGCC-3′) and subcloned first into pCAT (Ma and Reumann, 2008). The insert, EYFP-SKL was then, via pUC18ENTR2, further transferred into the plant expression vector pCAMBIA3300 via LR reaction for gene expression in BY-2 cells from the CaMV 35S promoter. pCAMBIA-EYFP-SKL was transformed into *Agrobacterium tumefaciens* GV3101::pMP90 (Hellens et al., 2000) by electroporation and kanamycin resistant clones were analyzed for the presence of the recombinant plasmid by restriction enzyme digest of the isolated plasmid DNA. Tobacco BY-2 cells were co-cultivated with the transgenic agrobacteria for 3 days and then spread out on blotting papers (MN 218 B, Macherey-Nagel, Düren, Germany) layered on top of MSMO medium (Sigma-Aldrich, Taufkirchen, Germany) agar plates containing 100 mg/l cefotaxim (Duchefa, Haarlem, The Netherlands). After 8 days the blotting papers with the cells were transferred to MSMO selection plates containing 100 mg/L cefotaxim and 0.3 mg/L bialaphos (Duchefa). Bialaphos resistant calli appeared after a 14-day incubation period on the selection plates. From these calli, suspension cell cultures were established and maintained as described (Schiermeyer et al., 2005). Consistent with previous reports, EYFP-SKL was efficiently targeted to peroxisomes with hardly any detectable cytosolic background staining (see **Figure 1**). One cell line with high EYFP expression level was selected for autophagy studies.

BY-2 transformants were cultured in MS growth medium at 25 ± 1°C with orbital shaking of 120 rpm in the dark. The medium consisted of MS basal salts with minimal organics (4.33 *g*/l), 3% (w/v) sucrose, 0.01% (w/v) myo-inositol, 0.2 mg/l 2,4-D, 1 mg/l thiamine hydrochloride, and 255 mg/l KH_2_PO_4_, pH 5.8. Under these culture conditions, the BY-2 cells reached the stationary phase after 6 days and were weekly sub-cultured (dilution 1:35 in 70 ml).

### AUTOPHAGY ANALYSIS DURING SUCROSE STARVATION

250 ml MS medium containing 3% sucrose was inoculated in duplicates with 4-day-old cells (1:25 dilution) and grown for 4 days. The cells were sedimented by centrifugation (100 × *g* for 5 min), washed twice with MS medium lacking sucrose and re-suspended in MS medium lacking sucrose ± 5 mM 3-MA. The cultures were incubated at 25 ± 1°C under rotation (120 rpm) in the darkness. During the 12-day period of analysis, three 1-ml aliquots (for determination of cell viability and EYFP fluorescence in living cells and for cytological observations) and one 15-ml aliquot [for determination of fresh weight (FW), protein content, and enzymatic activities] were taken from each culture daily.

### AUTOPHAGY ANALYSIS UNDER STANDARD GROWTH CONDITIONS

BY-2 cells were grown in MS medium containing 3% sucrose ± 5 mM 3-MA for 3 days. After sedimentation and washing, the cells were transferred back to standard MS medium (3% sucrose) lacking 3-MA and observed for additional 6 days (for 9 days altogether). 1-ml aliquots were taken daily for determination of cell viability and EYFP fluorescence in living cells and for light microscopy observations.

### PROTEIN EXTRACTION AND ANALYSIS OF PROTEIN CONTENT AND OF ENZYMATIC ACTIVITIES

The cells were sedimented by centrifugation, the residual MS medium was removed with a syringe, and the FW was determined. The cells were frozen in liquid N_2_, lyophilized and stored at –80°C. For extraction, the cells were supplemented with glass beads (800 μl beads Ø 0.25–0.50 mm and four beads Ø 0.4 cm) and 3 ml of extraction buffer [50 mM Hepes-KOH (pH 7.4), 5 mM MgCl_2_, 5 mM DTT, 2 mM benzamidin, 2 mM ε-amino-caproic acid, 1 mM Na_2_-EDTA, 1 mM EGTA, 0.5 mM PMSF, 0.1 % (v/v) Triton-X-100, 10 % (v/v) glycerol], vortexed (1 min) and incubated on ice (1 min). This procedure was repeated six times. After centrifugation (16000 *g*, 15 min, 4°C) the supernatant was frozen in small aliquots. The protein concentration was measured according to Lowry using bovine serum albumin as standard (Lowry et al., 1951). The activity of CAT was measured in 100 mM potassium phosphate buffer (pH 7.0) and 3% (v/v) H_2_O_2_ by monitoring H_2_O_2_ disproportionation at 240 nm (see Figure S1). The activities of fumarase and phosphoenolpyruvate carboxylase (PEPCx) were determined as described elsewhere (Stitt et al., 1978; Stitt, 1984). To investigate complete quenching of proteolytic activities in sucrose-starved BY-2 cells by protease inhibitors, proteins were extracted from BY-2 cells subjected to sucrose starvation for 0 and 6 days and the activities of two representative compartment-specific marker enzymes, PEPCx and CAT, were determined in separate and combined extract (mixed at a ratio of 1:1) after incubation on ice for 60 min. For the combined extract, theoretical activity values were calculated based on the data obtained from single extracts. For neither PEPCx nor CAT the measured enzymatic activities were significantly reduced (data not shown), indicating complete quenching of autophagy-induced proteolytic activities in protein extracts.

### CELL DEATH ASSAY

Accumulation of Evans’ blue in dead BY2 cells (living cells actively extrude the dye) was determined as in Takatsuka et al. (2004) and calculated as A_600_ per gram FW. 0% cell death was attributed to sucrose-supplied cells in the middle of logarithmic growth. To define 100% cell death, cells were killed by two cycles of freezing and thawing.

### INHIBITOR APPLICATION FOR CYTOLOGICAL STUDIES

3-MA and E-64 were applied as described by Takatsuka et al. (2004). ConcA was prepared as a 100 μM stock solution in DMSO and used at final concentration 1 μM. The cells supplied with inhibitors were cultured at 25 ± 1°C with rotation of 120 rpm. Aliquots were washed with 100 mM potassium phosphate buffer (pH 7.4) and analyzed by light microscopy.

### IMMUNOLOCALIZATION OF CATALASE

BY-2 transformants (200 μl of culture) were re-suspended in 600 μl 1% glutaraldehyde in MS medium, shaken vigorously for 15 min at room temperature (RT) and washed twice with MS medium. 400 μl solution containing 5 U/ml cellulase Onozuka R-10 (Serva), a mixture of 0.5 U/mg pectinase, 0.1 U/mg cellulase, 0.25 U/mg hemicellulase (Macerozyme R-10, Serva) and 5 U/ml pectinase (Fluka) dissolved in osmoticum (0.5% BSA, 0.01% β-mercaptoethanol, 50 mM CaCl_2_, 250 mM sorbitol, 100 mM Na acetate, pH 5.0) were added to the cells. Cells were incubated for 1 h at 28°C while shaking, washed three times with 600 μl PBS, then 600 μl of 0.3% Triton-X-100 in PBS were added and the cells were incubated for 15 min while shaking. After washing with 600 μl of 5% (w/v) BSA dissolved in PBS, cells were incubated in a new portion of 600 μl of 5% BSA solution for 1 h at RT (shaking). The cells were re-dissolved in 100 μl of anti-CAT antiserum (gift by Prof. Feierabend, University of Frankfurt, polyclonal rabbit antiserum against barley CAT) diluted 1:400 with 1% (w/v) BSA in PBS. Control cells were re-dissolved in 1% BSA solution. The cells were incubated at 4°C overnight, washed four times with PBS and incubated with Alexa 568-conjugated secondary antibody diluted 1:100 with 1% (w/v) BSA in PBS for 4 h in the dark at RT while shaking. The cells were washed four times with PBS and observed by confocal laser scanning microscopy (CLSM).

### WESTERN BLOTTING

After denaturation in Laemmli buffer, proteins were separated by SDS-PAGE and blotted to nitrocellulose membranes. After blocking of unspecific binding sites (1% dry milk powder in TBS buffer) the membranes were incubated in primary antiserum followed by secondary antibody. Primary antisera were kindly provided by Prof. Feierabend (University of Frankfurt, Germany, anti-CAT, polyclonal rabbit antiserum against barley CAT), Prof. Keegstra (MSU-DOE Plant Research Laboratory, East Lansing, MI, USA, anti-Toc75, polyclonal rabbit antiserum against pea Toc75), Prof. Feussner (University of Göttingen, Germany, polyclonal rabbit antiserum against cucumber isocitrate lyase, ICL), and Prof. Erdmann (University of Bochum, Germany, polyclonal rabbit antiserum against *Saccharomyces cerevisiae* Tom40). For detection of denatured EYFP a commercial monoclonal anti-GFP antiserum was used (Invitrogen). Protein dynamics were investigated by loading proteins from equal cell culture volumes. Western blot analysis by constant protein essentially showed the same dynamics as that relative to culture volume, but protein concentrations appeared to be overestimated at later stages of sucrose starvation, as shown by Coomassie Brilliant Blue staining (data not shown).

### QUANTIFICATION OF CELLULAR PEROXISOME NUMBERS

Individual BY-2 cells were separated from cell chains by addition of a similar volume of a mixture of cell wall-hydrolyzing enzymes dissolved in osmoticum as used in CAT immunolocalization protocol and incubation for 40 min at RT while shaking. The cells were fixed with 3.7% paraformaldehyde in 100 mM potassium phosphate buffer pH 7.4 and shaken at RT for 2 h to remove residual cell wall-hydrolyzing enzymatic activities. The cells were observed in potassium phosphate buffer by CLSM and cellular peroxisome numbers determined as described below.

### LIGHT AND CONFOCAL MICROSCOPY

A BX51 microscope (Olympus Deutschland GmbH, Hamburg, Germany) equipped with fluorescence and Nomarski DIC optics was used. Images were captured using a ColorView II digital camera and DP-Soft image-analytical software (Olympus Soft Imaging Solutions, Muenster, Germany). Peroxisomes were visualized by EYFP fluorescence using BP 450–480, DM 500, BA 515 filter. To visualize (auto)lysosomes, cells were incubated in 100 mM potassium phosphate buffer (pH 6.5) containing 1 μM Lysotracker Red (LTR, Invitrogen, Molecular Probes) at RT for about 5 min, spun down for 5 min, washed twice and re-suspended in the same buffer and observed using BP 545–580, DM 600, BA 610-IF filter. To visualize autophagosomes, cells were incubated in 100 mM potassium phosphate buffer (pH 6.5) containing 5 mM monodansylcadaverine (MDC, Sigma Aldrich) at RT for about 5 min, spun down for 5 min, washed twice and re-suspended in the same buffer and observed using BP 330–385, DM 400, BA 420 filter. In control (i.e., sucrose-supplied) cells, (auto)lysosomes (Figures S2A–D) were not detected.

Cellular peroxisome numbers were quantified by CLSM (Zeiss LSM 510, Carl Zeiss, Göttingen, Germany) using a 488/568-nm ArKr laser in combination with a 505- to 550-nm band-pass filter set. Z-stacks of optical sections were obtained throughout the individual cells. 3D projections were obtained for every Z-stack corresponding to a single cell using either ImageJ 1.37v software (NIH, USA), or AxioVision 4.7.2. software (Carl Zeiss, Germany) using ramp 50, maximum opacity 50, brightness 1 and threshold 10%. Peroxisomes were counted manually using cell counter tool. For immunolocalization of CAT, a 488/561-nm ArKr laser in combination with a 505- to 530-nm band-pass and 575-nm filter set was used.

### STATISTICS

The experiments were repeated at least three times each with similar results. The figures show data from a typical experiment if not otherwise indicated. The quantification of peroxisomes per cell was made for two sucrose starvation experiments and for two experiments with the growth of cells upon addition of 3-MA, respectively. For each variant, 10–18 individual cells were worked up, and mean numbers of peroxisomes per cell ± SE are shown. The significance of differences between cellular numbers of peroxisomes with vs. without addition of 3-MA at every time point of the experiments was analyzed using Student’s *t*-test.

## RESULTS

### STARVATION-INDUCED AUTOPHAGY REDUCES STEADY-STATE LEVELS OF PEROXISOMAL PROTEINS

To address peroxisome degradation by autophagy in tobacco BY-2 cells, we created stable transgenic lines expressing the peroxisome marker *EYFP-SKL* from the constitutive *CaMV 35S* promoter. The peroxisome marker was created by extending the fluorophore C-terminally by the C-terminal decapeptide (KALGLPVSKL>) of the *Arabidopsis* photorespiratory enzyme, hydroxypyruvate reductase. EYFP fluorescence was detected in numerous punctate subcellular structures (**Figures 1A–D**) that moved quickly within the cytosol. Organelle identity with peroxisomes was verified by immunocytochemical co-localization using a polyclonal antiserum against CAT (**Figures 1E–G**). CLSM confirmed that the strong targeting signal of hydroxypyruvate reductase EYFP-SKL (Ser-Lys-Leu) directed the fluorophore to peroxisomes with high efficiency, without any cytosolic background fluorescence when grown under nutrient-sufficient culturing conditions (**Figures 1E–F**). The *EYFP-SKL* lines did not show any growth phenotype compared to wild-type (WT) BY-2 cells, neither under sucrose-sufficient culturing conditions nor during sucrose starvation (data not shown).

**FIGURE 2:**
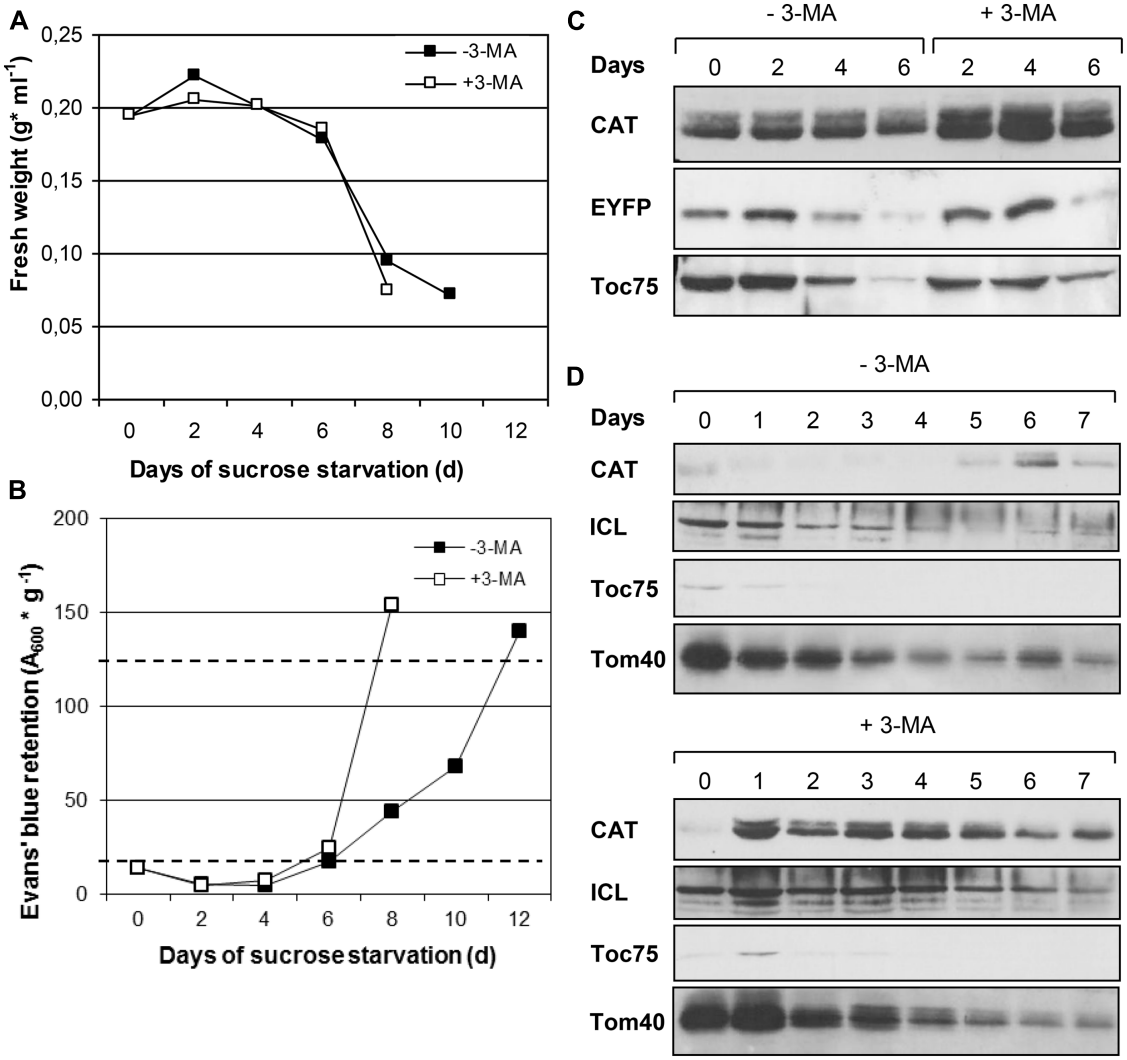
**Time course analysis of cell growth and organelle degradation in BY-2 transformants expressing *CaMV35S::EYFP-SKL* during sucrose starvation in the absence or presence of 3-MA.** Data from one representative experiment are shown. **(A)** Fresh weight. **(B)** Cell viability estimated as A_600__nm_ of Evans’ blue retained in dead BY2 cells relative to gram fresh weight. Full cell viability (0% cell death) and complete cell death (100%) corresponded to 20 OD units *g*^-1^ and 125 units *g*^-1^, respectively (dotted lines). **(C)** Protein levels of compartment-specific marker proteins CAT and EYFP (peroxisomes) and Toc75 (plastids) as determined by immunoblotting. The amounts of protein loaded in each lane corresponded to equal cell culture volumes **(C)** or protein amount (50 μg, **D**). The immunoblots of **(D)** stem from 3 large SDS-gels run, transferred, incubated and developed in parallel. Due to cutting of the large membranes into two halves for control and 3-MA-treated cells, the lanes are not presented continuously.

Since autophagy is reported being induced in BY-2 cells upon nutrient starvation (Moriyasu and Ohsumi, 1996), we first investigated whether peroxisomes are degraded by autophagy under sucrose starvation. When cultured to mid-log phase under nutrient-sufficient conditions and thereafter deprived of sucrose, the BY-2 cells largely stopped cell growth and proliferation, as indicated by constant FW (**Figure 2A**). Despite nutrient starvation, the cells remained viable for about 6 days, as determined by Evans’ Blue dye exclusion assay, indicating the activity of an efficient endogenous mechanism for cell remodeling and nutrient recycling such as autophagy. Subsequently, the cells slowly started dying (>day 8), coinciding with a drastic decline in FW (**Figures 2A,B**). Hence, heterotrophic BY-2 cells cultured without external carbon source can survive for nearly 1 week by using endogenous energy resources such as internal stores and/or autophagic recycling mechanisms. Application of the autophagy inhibitor 3-MA dramatically accelerated cell death (**Figure 2B**), consistent with the reported function of autophagy in cellular remodeling and survival during nutrient starvation in BY-2 cells (e.g., Takatsuka et al., 2004; Toyooka et al., 2006). Hence, the overexpressor lines with EYFP labeled peroxisomes showed similar growth characteristics and responses to sucrose starvation compared to WT, thereby allowing autophagic degradation studies of peroxisomes *in vivo*.

We first investigated peroxisome dynamics by biochemical means, applying immunoblotting with polyclonal antibodies against two endogenous and one recombinant soluble peroxisomal matrix proteins, CAT, ICL, and EYFP-SKL. Upon sucrose starvation the levels of the three peroxisomal matrix proteins decreased during the following 6 days, indicating proteolytic degradation of peroxisomal matrix protein (**Figures 2C,D**). In the presence of 3-MA, the three matrix proteins showed higher protein levels compared to culturing conditions in inhibitor absence. This effect was most pronounced for the native peroxisomal matrix proteins (CAT and ICL) at various time points and for the recombinant protein (EYFP-SKL) after 4 days of sucrose starvation (**Figures 2C,D**). Interestingly, the chosen marker proteins for plastids (Toc75) and mitochondria (Tom40) showed a similar trend but only a marginal effect of 3-MA (**Figures 2C,D**), indicating that chloroplasts and mitochondria were degraded by starvation-induced autophagy to much lesser extent.

We complemented the analyses of organelle dynamics by activity analyses of compartment-specific marker enzymes such as CAT, fumarase (mitochondria), and PEP carboxylase (cytosol). Of several starvation experiments showing the same trend, one representative example is shown. The activities of all marker enzymes declined during sucrose starvation with minor differences in kinetics (Figure S1). In the presence of 3-MA the activities of the organelle marker enzymes remained higher and the starvation-induced loss of enzymatic activity was delayed (Figures S1B–D). The simplest explanation for the higher marker activities of 3-MA samples in Figures S1B–D is that they likely resulted from higher levels of enzyme protein in 3-MA-treated cells (Figure S1A). Along the same line, the total cellular protein content declined during sucrose starvation, and this effect was inhibited by 3-MA (Figure S1A). Control experiments verified that the decrease in protein content and enzymatic activities was not reduced *in vitro* by high proteolytic activities of sucrose-starved cells (data not shown; see Materials and Methods).

### MICROSCOPIC ANALYSIS OF PEROXISOMES AND AUTOPHAGIC ORGANELLES

**FIGURE 3:**
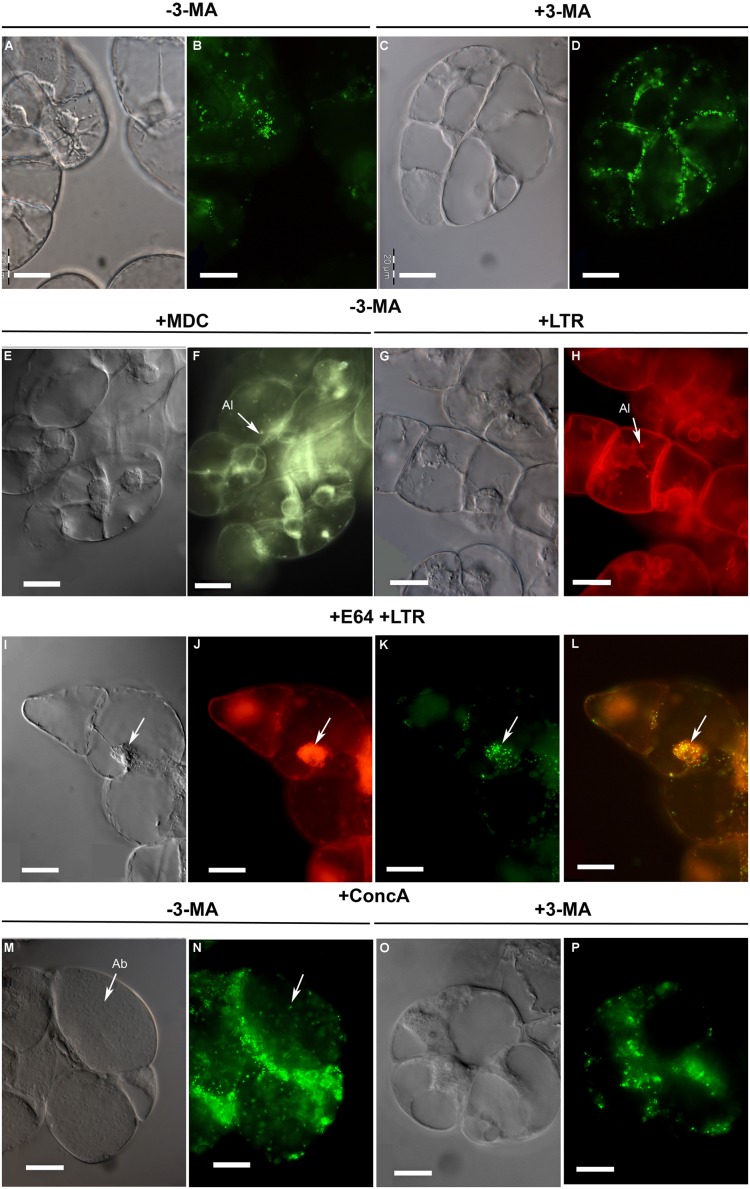
**Microscopic analysis of peroxisome dynamics during sucrose starvation. (A–D)** BY-2 cells were subjected to sucrose starvation for 2 days in the absence (**A,B**; –3-MA) or presence of 3-MA (**C,D**; +3-MA). Note peroxisome clustering around the cell nucleus in the absence of 3-MA (at this magnification seen as green clots in the cell center). **(E–H)** Monodansylcadaverine (MDC)-positive organelles **(E,F)** and LysoTracker red (LTR)-positive organelles **(G,H)**, both indicative of autolysosomes (Al), appeared in the cells and were detected by staining after sucrose starvation (32 h) in the absence of 3-MA. **(I–L)** To achieve autolysosome accumulation, BY-2 cells were subjected to sucrose starvation alone for 8 h and in the presence of E-64 for an additional 24 h. The arrow points to a subcellular aggregate of putative autolysosomes **(I,J)** around the cell nucleus and peroxisomes **(K)** with image overlay (L = J+K). **(M–P)** After 2 days of sucrose starvation and in the presence of ConcA autophagic bodies (Ab, in **M**) and EYFP fluorescent structures **(N)** were detected in the central vacuole **(M,N)**. **(O,P)** Application of 3-MA inhibited ConcA-dependent autophagic body formation **(O)** and the appearance of EYFP fluorescent structures in the central vacuole **(P)**. Images were taken with Nomarski optic **(A,C,E,G,I,M,O)** and epifluorescence **(B,D,F,H,J,K,N,P)**. Scale bar: 20 μm.

Consistent with the biochemical data (see above), the cellular abundance of peroxisomes gradually declined with the duration of sucrose starvation. For instance, after 2 days of sucrose starvation the cellular number of peroxisomes was visibly reduced as compared to sucrose-supplied cells (**Figures 3A,B**; compare to **Figures 1B,D**), indicative of peroxisome turnover. Interestingly, also the shape and subcellular localization of EYFP-labeled peroxisomes changed. While the peroxisomes of BY2 cells cultured under nutrient-rich conditions were rather evenly distributed in the cytosol (**Figures 1B,D**), those of sucrose-starved cells tended to cluster around the nucleus (**Figures 3A,B**). In the presence of 3-MA, the cellular number of peroxisomes was visibly higher, further supporting the conclusion that peroxisomes are degraded by autophagy. Interestingly, peroxisome clustering around the nucleus was not observed in 3-MA-treated cells (**Figures 3C,D**), suggesting that the change in subcellular peroxisome localization preceded autophagic peroxisome degradation and was triggered by the signal transduction cascade inducing autophagy.

**FIGURE 4:**
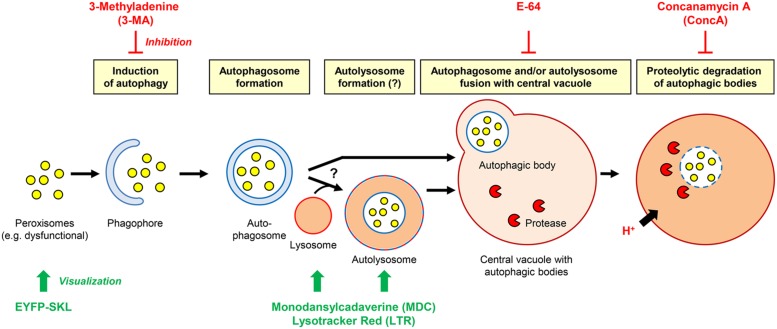
**Hypothetical working model of the degradation of plant peroxisomes in tobacco by autophagy.** The effect of inhibitors and method of organelle visualization are indicated.

In tobacco cells treated with protease inhibitors, cytosolic structures to be degraded by autophagy are sequestered in autophagosomes that are subsequently converted to autolysosomes and release their content as autophagic bodies into the vacuole (Bassham, 2007, 2009). The results above suggested an analogous mechanism for autophagic peroxisome degradation in tobacco BY2 cells (**Figure 4**). Autophagy can be analyzed by staining with the acidotropic fluorescent dye monodansylcadaverine (MDC), which labels autolysosomes and possibly other acidic organelles (**Figure 4**; Contento et al., 2005). Due to their short half-life in the absence of inhibitors, autolysosomes can generally only be observed in the presence of inhibitors that block downstream steps of autophagic organelle processing (Bassham, 2007, 2009). Indeed, when staining sucrose-starved BY-2 cells with MDC (in the absence of 3-MA and E-64), only very few punctate structures could be visualized (**Figure 3F**). Likewise, only few LysoTracker Red (LTR)-positive organelles, which might represent (auto-)lysosomes (Al), could be visualized in sucrose-starved BY-2 cells (in the absence of 3-MA and E-64, **Figure 3H**). This result is consistent with the high turnover rate of autolysosomes.

To provide further evidence that peroxisomes are degraded by autophagy and to investigate a possible accumulation of autophagic vesicles, we applied the cysteine protease inhibitor, E-64, which blocks the transfer of autolysosomes to the central vacuole (**Figure 4**) and leads to pronounced accumulation of autolysosomes in the cytosol (Moriyasu and Inoue, 2008). Indeed, E-64 caused a drastic increase in the cellular abundance of LTR-stained organelles, particularly in the initial phase of sucrose starvation (32 h, **Figure 3J**). Interestingly, the LTR-positive organelles often formed large subcellular clusters in close proximity to the nucleus (**Figures 3I,J**). In parallel, the peroxisomes clustered in the same cell areas as the LTR-stained organelle aggregates, suggesting that peroxisomes were degraded inside autolysosomes (**Figures 3K,L**). After 5 days of sucrose starvation, E-64 no longer caused accumulation of autolysosomes, even upon prolonged application time (48 h, data not shown). The data suggested that autophagy ceased after about 4 days of starvation in our experimental system and was replaced by a yet unknown mechanism of cell death.

When vacuolar degradation of autophagic bodies was blocked by the vacuolar V-ATPase inhibitor, ConcA, the central vacuole became filled with small particles (**Figure 3M**). As concluded previously (Thompson et al., 2005), the vacuolar structures most likely represented autophagic bodies (Ab). If this were the case, the accumulation of these bodies in the central vacuole should depend on the induction of autophagy and be abolished when blocking the signal transduction pathway inducing autophagy by 3-MA. As expected, the simultaneous application of both ConcA and 3-MA prevented the accumulation of particles in the vacuole, strengthening the idea that these organelles represented autophagic bodies (**Figures 3O,P**). The ConcA-dependent accumulation of vacuolar autophagic bodies was paralleled by the appearance of yellow fluorescent punctuate structures in the central vacuole (**Figure 3N**), suggesting that some vacuolar autophagic bodies contained peroxisomes and their remnant matrix proteins including EYFP-SKL. A certain degree of uniform background fluorescence of the vacuolar lumen was also observed, consistent with execution of the subsequent steps of autophagy when autophagic bodies and their organellar content become dissolved (**Figure 4**). Similar to E-64, ConcA ceased causing detectable accumulation of autophagic bodies in the vacuoles of the cells at advanced stages of starvation (data not shown).

### REDUCTION OF CELLULAR PEROXISOME NUMBERS BY STARVATION-INDUCED AUTOPHAGY

**FIGURE 5:**
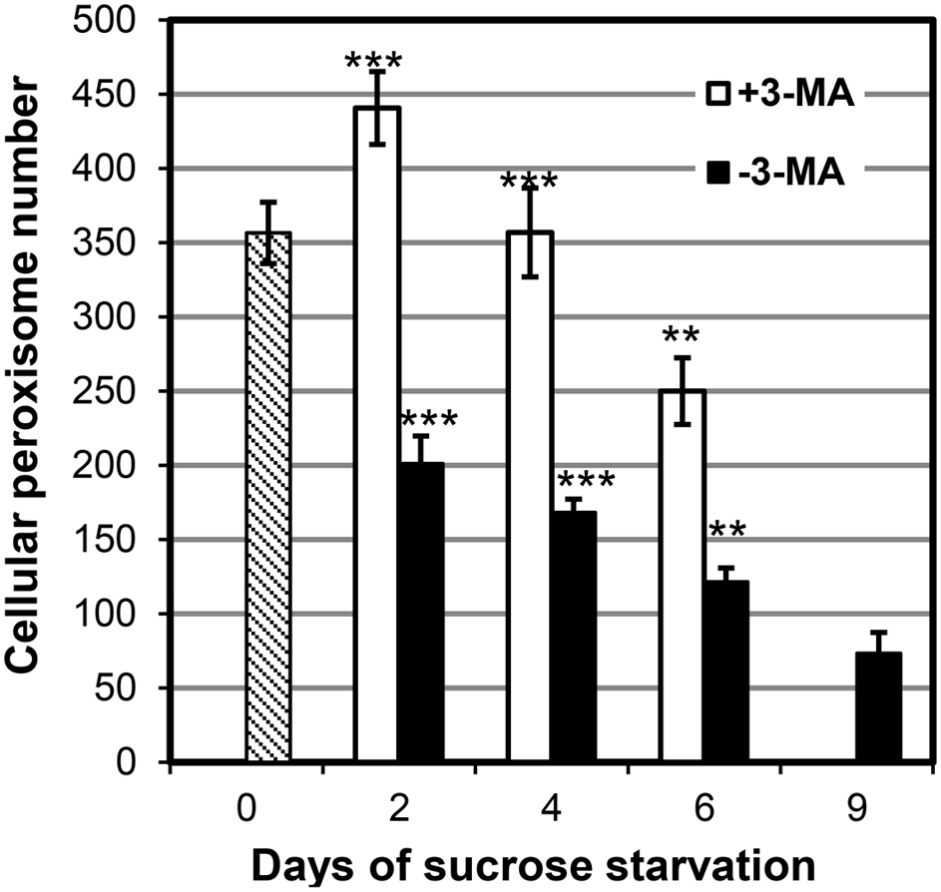
**Quantification of peroxisome dynamics in BY-2 EYFP-SKL cells under sucrose starvation by CLSM in the absence or presence of 3-MA.** Data from one representative experiment are shown. For each time point, 10–18 individual cells were analyzed. Mean values of peroxisome numbers per cell ± SE are shown. Stars indicate significant differences between –3-MA and +3-MA samples at the same timepoint (***p < 0.0001, **p < 0.001).

To investigate the effect of starvation-induced autophagy on BY2 cell peroxisomes in greater details, we determined cellular peroxisome numbers quantitatively by CLSM in a time course experiment in the absence and presence of 3-MA. BY-2 cells grown to mid logarithmic phase in nutrient-rich conditions (3% sucrose) contained on average about 350 peroxisomes per cell (day 0, **Figure 5**). Upon autophagy induction by sucrose starvation, the average cellular peroxisome number of control cells (i.e., in the absence of 3-MA) decreased by ∼40% (to about 200, day 2) within the first 2 days and by ∼70% (to 120 peroxisomes per cell) by day 6. The cellular steady-state number of peroxisomes is determined by the rates of peroxisome biogenesis and degradation and cell division. The results suggest that at least 70% of cellular peroxisomes were degraded, and even more if peroxisome proliferation continued.

When starvation-induced autophagy was blocked by 3-MA, the cellular number of peroxisomes first increased from 350 (day 0) to ∼440 (day 2) and declined thereafter. Hence, peroxisome proliferation continued (possibly at lower pace compared to nutrient-sufficient conditions) in the first days of starvation in the presence of 3-MA. Compared to sucrose-starved control cells, the blockage of autophagy led to a ∼2-fold increase in cellular peroxisome number (day 2). Similarly, at later stages of sucrose starvation (e.g., 4 and 6 days) the cellular peroxisome number was ∼2-fold higher in cells treated with 3-MA as compared to the controls. Taken together, the decreasing steady-state levels of cellular peroxisomes in sucrose-starved BY2 cells and the 2-fold accumulation of peroxisomes in cells unable to execute starvation-induced autophagy further supported the previous pharmacological, biochemical, and microscopic data that whole tobacco BY2 cell peroxisomes are degraded by starvation-induced autophagy.

Interestingly, in the presence of 3-MA the cellular peroxisome number did not remain constant but decreased steadily (day 2: 440, day 4: 360, day 4: 250, **Figure 4**). Since the cells remained fully viable (**Figure 2B**), the data indicate that either starvation-induced peroxisome degradation was incompletely blocked or that it was complemented by another mechanism different from autophagy. The latter explanation is in line with the observations that autophagy-specific structures could no longer be detected after the fourth day of starvation (see above). This second mechanism for peroxisome degradation might be induced on top of autophagy at late stage of sucrose starvation.

### PEROXISOME DEGRADATION BY CONSTITUTIVE AUTOPHAGY

**FIGURE 6:**
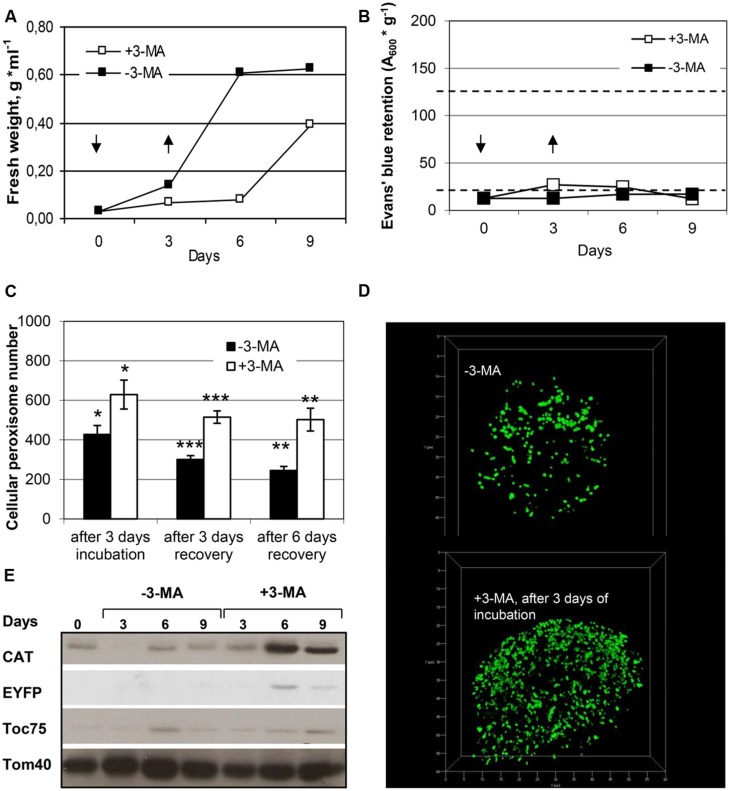
**Quantification of peroxisome dynamics in BY-2 cells under standard growth conditions.** Tobacco BY-2 EYFP-SKL cells were cultivated under nutrient-rich standard growth conditions (3% sucrose) and in the absence or presence of 3-MA and analyzed for **(A)** fresh weight, **(B)** cell viability, **(C)** cellular peroxisome number (mean values of peroxisome numbers per cell ± SE are shown; stars indicate a significant difference between –3-MA and +3-MA samples at the same timepoint: ***p < 0.0001, **p < 0.001, *p < 0.05) as determined from 3D-projections (examples shown in **D**), and **(E)** protein levels of compartment-specific markers CAT (peroxisomes), Toc75 (plastids) and Tom40 (mitochondria). In the absence of 3-MA, the signals for CAT (3 days) and EYFP were below the detection limit in the given experiment. The amounts of protein loaded into each lane correspond to equal volumes of cell culture. Full cell viability (0% cell death) and complete cell death (100%) corresponded to 20 OD units g^-1^ and 125 OD units *g*^-1^, respectively (dotted lines in **C**). Arrows indicate the time points of 3-MA application and removal by cell washing. Data from one representative experiment are shown.

Given that peroxisomes are a major site of cellular ROS production, their matrix proteins are prone to oxidative damage, and efficient elimination of dysfunctional plant peroxisomes might be required under standard growth conditions. Recent plant autophagy studies in *Arabidopsis* support this idea (Shibata et al., 2013; Yoshimoto et al., 2014). To address constitutive peroxisome turnover in BY-2 cells, we investigated peroxisome degradation under nutrient-rich conditions. Tobacco BY-2 cells were first grown to mid-log phase and low cell density in the presence of sucrose (3%), then the medium was removed by gentle centrifugation, and the cells were re-suspended in a similar volume of fresh medium (day 0), either in the absence or presence of 3-MA. After 3 days the inhibitor was removed by multiple cell washes and the cells were transferred to new nutrient-rich MS medium (3% sucrose) lacking 3-MA (day 3) and analyzed after additional three to 6 days (day 6 and day 9, respectively). Control cells grown in the absence of 3-MA were sub-cultured identically except for the omission of 3-MA. As shown by FW analysis, BY-2 cells not exposed to 3-MA grew at exponential rate in sucrose-containing growth medium and reached stationary phase approximately at day 6, when minimal signs of cell death became detectable (**Figures 6A,B**). The blockage of (constitutive) autophagy by 3-MA negatively affected BY-2 cell proliferation (FW, **Figure 6A**). Cell proliferation largely stagnated but after inhibitor removal (day 3), the cells recovered, as indicated by nearly 2-fold daily increase in FW (**Figures 6A,B**). In the representative experiment shown (**Figure 6B**), the rate of cell death 3 days after 3-MA addition was 8%; in other experiments, however, it was reaching ∼20% (data not shown).

To investigate peroxisome dynamics under nutrient-rich conditions, we determined cellular peroxisome numbers as describe above. The data yielded two major results. First, steady-state levels of cellular peroxisome numbers decreased with culture age and progressing cell senescence, i.e., from early exponential to late stationary phase. While BY-2 cells contained on average about 400 peroxisomes per cells at early exponential growth phase (day 3), the number decreased to about 300 at early (day 6) and 250 at late stationary phase (day 9). Notably, the extent of cell death was only marginal under these conditions (<10%, **Figure 6**).

Second, 3-MA caused a clearly visible increase in the cellular number of peroxisomes which was statistically significant after three and 6 days of recovery (**Figures 6C,D**). The average cellular peroxisome number was about 1.7 (after 3 days of recovery) to 2.0-fold higher (after 6 days of recovery) in BY-2 cells exposed transiently to 3-MA, even though grown under nutrient-rich conditions, as compared to control cells. Since control and treated cells were affected differently with respect to cell proliferation in this experiment (contrary to sucrose starvation), as indicated by exponentially increasing FW (for control cells) and stagnant FW (for 3-MA treated cells), not only the blockage of peroxisome degradation but also continuous of peroxisome proliferation might have affected the 2-fold higher cell numbers of peroxisomes in MA-treated cells (see Discussion).

The inhibitory effect of 3-MA on peroxisome degradation was supported by immuno-biochemical analysis of CAT protein levels, which clearly increased upon cell exposure to 3-MA. Interestingly and consistent with the starvation data, among the three major cell organelles investigated, the effect of 3-MA appeared to be most pronounced for peroxisomes (**Figure 6E**). Taken together the data indicated that peroxisomes are degraded even under nutrient-sufficient conditions and at early exponential phase to considerable extent by constitutive autophagy.

## DISCUSSION

Recently, several research groups independently published conclusive evidence that *Arabidopsis* peroxisomes are degraded by autophagy and that this process is important for quality control of peroxisomes (Farmer et al., 2013; Kim et al., 2013; Shibata et al., 2013; Yoshimoto et al., 2014). While *Arabidopsis* research benefits from public collections of knock-out mutants, which lack for other plant species, tobacco BY-2 cells offer a complementary model system with several advantages to study plant autophagy in general and pexophagy in particular. First, autophagy can easily be induced in heterotrophic suspension-cultured cells by omission of essential nutrients in the growth medium (Moriyasu and Ohsumi, 1996; Takatsuka et al., 2004). The uniform cell type further standardizes cellular effects. Second, pharmacological inhibitors of autophagy that target key component of the autophagic machinery but are difficult to apply to entire *Arabidopsis* plants, are rapidly taken up by BY-2 cell lines and efficiently carry out their inhibitory functions. Third, the cellular peroxisome pool can be quantified relatively straight-forward in suspension-cultured cells. This analytical tool can be further explored in future quantitative studies of plant pexophagy addressing, for instance, which signal processes contribute to peroxisome turnover and which molecular players are involved (receptors, etc.). Last, stable transgenic tobacco cell lines with specific labeling of cell organelles by fluorescent markers can be generated.

Our investigations of peroxisome degradation are based on stable transgenic lines with fluorescently labeled peroxisomes expressing *EYFP-SKL*. Due to the very strong targeting signal of hydroxypyruvate reductase, the reporter protein was targeted quantitatively to peroxisomes (**Figure 1**), thereby facilitating monitoring of peroxisomes with high microscopic and biochemical detection sensitivity. The high proteolytic stability of the β-barrel protein of GFP variants allowed autophagic pathway analysis until the very last stages in the vacuole (**Figures 3** and **5**) and quantitative determination of peroxisome degradation by EYFP immunoblotting analysis and cellular EYFP fluorescence. The reporter protein data were confirmed for two native plant peroxisomal marker proteins (CAT and ICL, **Figures 2** and **6**). The independent analytical methods complemented each other and allowed the underlying mechanism to be characterized as macroautophagy. The kinetics of protein accumulation differed slightly for three peroxisomal matrix proteins, and 3-MA-dependent protein accumulation was most pronounced for CAT (**Figures 2C,D** and **6E**). The data might suggest that particularly peroxisomes with a high content of CAT (possibly including oxidatively damaged CAT protein) are specifically prone to autophagic degradation, which needs to be investigated in greater detail in future studies. Kim et al. (2013) showed that even for two glyoxylate cycle enzymes, ICL and malate synthase, the kinetics of protein accumulation differed in mutants compromised in autophagy (*atg7-2* and *atg5-1*, Figure 2 in Kim et al., 2013).

Recent studies focused on autophagic degradation of peroxisomes (obsolete or damaged) in plant cells under normal conditions, i.e., in course of “constitutive,” or “basal,” autophagy (Inoue et al., 2006; Farmer et al., 2013; Kim et al., 2013; Shibata et al., 2013; Yoshimoto et al., 2014). They showed that constitutive autophagy is an important mechanism of the quality control of peroxisomes, which interacts with peroxisomal protein degradation by Lon2 protease in a tightly regulated manner (Farmer et al., 2013; Goto-Yamada et al., 2014a,b). Constitutive autophagy is active also in the absence of any stresses and is essential for plant survival (Inoue et al., 2006).

In our study we focused on degradation of peroxisomes during bulk autophagy-induced by carbohydrate starvation. Starvation of carbon (e.g., low light intensity) or nitrogen (insufficient nitrate and ammonium availability) are common abiotic stress conditions plants have to cope with in their natural environment. We demonstrate in this study that starvation-induced autophagy has a pronounced effect on peroxisome biology and induced pexophagy in BY-2 cells. Peroxisome degradation by autophagy was confirmed by several lines of evidence. First, the total number of cellular peroxisomes decreased particularly in the first phase of sucrose starvation (**Figure 5**). Second, this process was inhibited by 3-MA which has been previously described to block autophagy in BY-2 cells (Moriyasu and Ohsumi, 1996; Takatsuka et al., 2004; Moriyasu and Inoue, 2008). Even though 3-MA, which inhibits class I as well as class III phosphatidylinositol 3-kinases (Klionsky et al., 2008), is not fully specific to autophagy but also reported to inhibit, for instance, cell growth, multiple lines of evidence and indications obtained in former and this study strongly suggest that the major primary effect of 3-MA application on sucrose-starved BY-2 cells was the inhibition of autophagy including peroxisome degradation. Quantification of cellular peroxisome number by confocal microscopy provided solid evidence that 3-MA led to a significant increase in cellular peroxisome numbers and blocked peroxisome degradation in sucrose-deprived cells.

Third, upon application of inhibitors that block macroautophagy at specific stages such as autolysosome acidification and vesicle fusion with the vacuole (E-64, Yamada et al., 2005) and autophagic body degradation (ConcA), autolysosomes and autophagic bodies were shown to coincide with peroxisomes, strongly suggesting that the autolysosomes carried peroxisomes as autophagic cargo. Since acidotropic dyes such as MDC and LTR may also stain acidic organelles other than autolysosomes (Klionsky et al., 2012; Merkulova et al., 2014), our cytological studies do not allow conclusive identification of the acidic organelles as autolysosomes. More comprehensive cytological analyses, for instance, by labeling autophagosomes with a fluorescent reporter attached to ATG8 need to extend this first study of peroxisome degradation in tobacco. Taking all data of this study together, conclusive evidence is provided that peroxisomes are degraded in sucrose-starved tobacco BY-2 cell by autophagy.

Investigations of peroxisome degradation in unicellular fungi and mammals focused on superfluous peroxisomes in the past. Since peroxisome proliferation can be easily induced by external stimuli (e.g., MeOH, fatty acids, clofibrate) in these organisms, the degradation of superfluous peroxisomes can be subsequently analyzed by removal of the chosen stimulus. In attempts to similarly induce peroxisome proliferation in tobacco BY-2 cells, we investigated the effects of various components such as fatty acids, detergents (Tween series), bezafibrate (a known peroxisome proliferator in mammalian cells), and ROS (e.g., H_2_O_2_) on peroxisome abundance. However, the stimuli were either not compatible with BY-2 cell viability or did not affect peroxisome abundance (data not shown). Besides, it remains unknown whether plant peroxisomes become superfluous under any physiological conditions.

The physiological function of constitutive autophagy is most likely the elimination and recycling of protein aggregates and dysfunctional organelles whose matrix enzymes and membrane lipids have been oxidatively and irreversibly damaged (Komatsu et al., 2007a). Consistent with these data, in BY-2 cells grown under nutrient-rich conditions, 3-MA caused a 1.5- to 2-fold accumulation of peroxisomes within 3 days (**Figure 6**). However, since steady-state levels of organelles are determined by three parameters, i.e., organelle biogenesis, organelle degradation and cell proliferation, the results are more difficult to interpret. Cell proliferation was differentially affected in control and 3-MA treated cells, as indicated by exponentially increasing FW of control cells and stagnant FW of 3-MA treated cells. Hence, continuous peroxisome proliferation in the absence of cell division might have caused an increase in cellular numbers of peroxisomes in MA-treated cells. More detailed analyses of cell and peroxisome proliferation are needed to decipher the underlying causes of the observed results.

The blockage of constitutive peroxisome degradation by 3-MA was further supported by immunnobiochemical data. CAT protein strongly and EYFP-SKL moderately accumulated in 3-MA treated cells. This result strongly suggests that degradation of peroxisomes occurs by constitutive macroautophagy under standard growth conditions. Interestingly, 3-MA did not cause a comparable accumulation of marker proteins of other cellular compartments like plastids and mitochondria (**Figure 6E**). This suggests that the turnover rate of peroxisomes is highest amongst the major metabolic cell organelles. Since the total cellular protein content of peroxisomes is far lower than that of plastids, these results indicate that the function of peroxisome degradation is the elimination of dysfunctional organelles rather than recycling of proteins for cellular remodeling.

The need for pronounced peroxisome degradation under standard growth conditions can be rationalized by the organelle’s oxidative metabolism. Xiong et al. (2007a,b) showed that ROS can induce macroautophagy in *Arabidopsis*. Macroautophagy-defective RNAi-AtATG18a transgenic plants were more sensitive to ROS and accumulated high levels of oxidized proteins due to a lower rate of protein degradation, indicating that autophagy is involved in degrading oxidized proteins under both oxidative stress and normal growth conditions (Xiong et al., 2007a,b). Peroxisomes are a compartment of high ROS production by various metabolic pathways including photorespiration (Foyer and Noctor, 2003), fatty acid β-oxidation, polyamine and cofactor metabolism (Reumann, 2011), which might explain the especially high oxidative damage for these organelles also in heterotrophic BY-2 cells.

While starvation-induced peroxisome degradation is expected to target different peroxisome populations rather unspecifically, it is reasonable to hypothesize that constitutive peroxisome degradation specifically selects “old/senescent” and dysfunctional peroxisomes for elimination (**Figure 4**). Future studies need to address identification of the signal(s) emitted from dysfunctional and/or senescent peroxisomes and the components of signal transduction cascades activating constitutive autophagy under standard growth conditions. Hydrogen peroxide leaking from dysfunctional peroxisomes due to inactivation of oxidatively damaged CAT or reduced permeability of the peroxisomal membrane due to lipid peroxidation is the predicted candidate for the signaling molecule (Shibata et al., 2013; Goto-Yamada et al., 2014a,b). The mechanism responsible for specific labeling of dysfunctional peroxisomes might involve p62/SQSTM1, as demonstrated for mammalian cells (Kaniuk et al., 2007; Komatsu et al., 2007b; Pankiv et al., 2007; Kim et al., 2008).

In addition, several independent lines of indications emerged from this study that peroxisomes might be degraded, in addition to autophagy, by a complementary mechanism. First, even in the presence of 3-MA the average cellular peroxisome number steadily decreased under starvation conditions (**Figure 5**). Since relatively high inhibitor concentrations were used (5 mM) and 3-MA is not reported to be instable, it is unlikely that the inhibitory effect 3-MA ceased at advanced starvation stage. Furthermore, while peroxisome degradation continued (**Figure 5**), neither E-64 nor ConcA caused autolysome or autophagic body accumulation, respectively, at this advanced stage of sucrose starvation (data not shown). Apparently, autophagosomes and autolysosomes were no longer formed on a larger scale. The activity of macroautophagy seemed to decline and the catabolic process be replaced by another degradation mechanism. Notably, the extent of cell death still remained rather low in the absence of 3-MA. At late stage of sucrose starvation, some peroxisomes appeared to disassemble directly in the cytosol. Future work needs to address the identity of this second non-autophagic degradation mechanism of peroxisomes.

## CONCLUSION

In conclusion, we demonstrated in the present study that (i) peroxisomes are continuously degraded in tobacco BY-2 cells not only under nutrient starvation but also under optimal growth conditions, (ii) that these two processes are mediated by the machinery and mechanism of autophagy, (iii) that the extent of constitutive peroxisome turnover is considerable and exceeds that of other cell organelles (plastids, mitochondria), and (iv) that a second mechanism is likely to act on top of autophagy at advanced stages of nutrient starvation and cell senescence. We established a new model system for cell biological analyses of plant peroxisomes using tobacco BY-2 suspension-cultured cells. The system has been proven ideal for pharmacological and microscopic analyses, complements genetic studies in *Arabidopsis* and will assist in future advanced molecular analyses of plant pexophagy.

## Conflict of Interest Statement

The authors declare that the research was conducted in the absence of any commercial or financial relationships that could be construed as a potential conflict of interest.

## ABBREVIATIONS

Ab: autophagic body
Al: autolysosome
ATG: autophagy-related gene
BY-2: Nicotiana tabacum L. cv. Bright Yellow 2
CaMV: cauliflower mosaic virus
CAT: catalase
CLSM: confocal laser scanning microscopy
ConcA: concanamycin A
DEHP: di(2-ethylhexyl) phthalate
DIC: differential interference contrast
DMSO: dimethylsulfoxide
EYFP: enhanced yellow fluorescent protein
FW: fresh weight
ICL: isocitrate lyase
LTR: Lysotracker Red
MDC: monodansylcadaverine
3-MA: 3-methyladenine
MS: Murashige and Skoog
OD: optical density
PEX: peroxisomal biogenesis protein
PEPCx: phosphoenolpyruvate carboxylase
RT: room temperature
ROS: reactive oxygen species
