# Supplementary material for: Plant peroxisomes are degraded by starvation-induced and constitutive autophagy in tobacco BY-2 suspension-cultured cells
Source: Front Plant Sci. 2014 Nov 18;5:629. doi: 10.3389/fpls.2014.00629 (PMC4235271; doi:10.3389/fpls.2014.00629)
Supplement: Supplementary file 1 [file Data_Sheet_1.DOC]

***Supplementary Material***

**Plant peroxisomes are degraded by starvation-induced and constitutive autophagy in tobacco BY-2 suspension cultured cells**

**Olga V. Voitsekhovskaja^1, 2^, Andreas Schiermeyer ^3^, and Sigrun Reumann ^1, 4^**^*^

^1^ Georg-August-University of Goettingen, Albrecht-von-Haller-Institute for Plant Sciences, Department of Plant Biochemistry, Goettingen, Germany

^2^ Komarov Botanical Institute, Russian Academy of Sciences, Plant Ecological Physiology, St. Petersburg, Russia

^3^ Fraunhofer-Institut für Molekularbiologie und Angewandte Oekologie, Abteilung Pflanzenbiotechnologie, Aachen, Germany

^4^ Faculty of Science and Technology, Centre for Organelle Research (CORE), University of Stavanger, Stavanger, Norway

^5^ Faculty of Mathematics, Informatics and Natural Sciences, Biocentre Klein Flottbek, University of Hamburg, Hamburg, Germany

*** Correspondence:** Sigrun Reumann, Faculty of Mathematics, Informatics and Natural Sciences, Biocentre Klein Flottbek, University of Hamburg, Ohnhorststrasse 18, D-22609 Hamburg, Germany; Phone: +49(0)40/42816-743, E-mail:  [sigrun.reumann@uni-hamburg.de](mailto:sigrun.reumann@uni-hamburg.de)

## Supplementary Figures


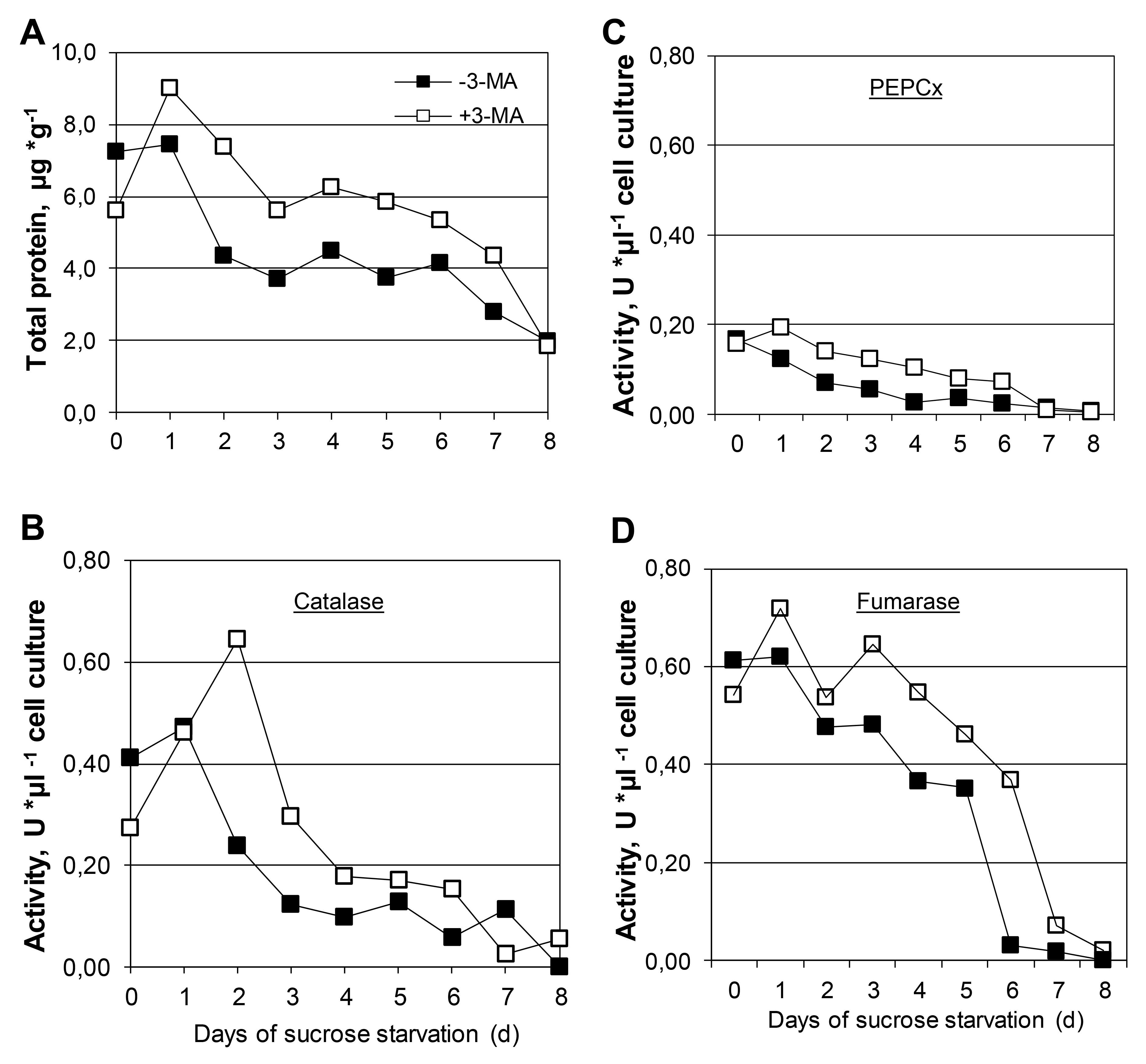


Supplementary Figure 1. Time course analysis of compartment-specific marker enzyme activities in BY-2 cells during sucrose starvation. (A) Relative protein content per gram fresh weight. (B – D) enzymatic activities of marker enzymes CAT (peroxisomes, B), PEP-Cx (cytosol, C), and fumarase (mitochondria, D), expressed in U per µl cell culture volume. Data from one representative experiment are shown. Each time point represents the mean value ±SE of two technical replicates. (A-D), bars are smaller than the symbols.


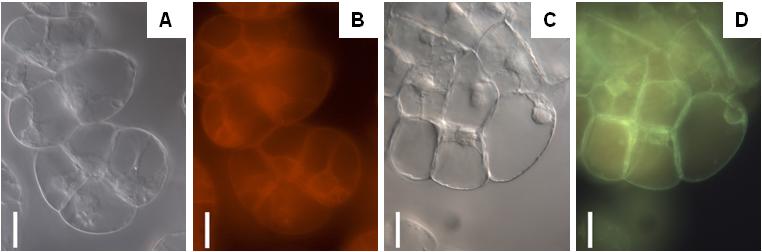


Supplementary Figure 2. Staining of control BY-2 cells grown under nutrient-sufficient conditions. BY-2 cells grown in the presence of 3% sucrose did not show lysosomes when stained with LysoTracker Red (LTR, B) or monodansylcadaverine (MDC, D). A, C, Nomarski optics; B, staining with LTR; D, staining with MDC. Scale bar: 20 µm
